# Spatial and temporal boundaries of NMDA receptor hypofunction leading to schizophrenia

**DOI:** 10.1038/s41537-016-0003-3

**Published:** 2017-02-03

**Authors:** Kazu Nakazawa, Vivek Jeevakumar, Kazuhito Nakao

**Affiliations:** Department of Psychiatry and Behavioral Neurobiology, University of Alabama at Birmingham, Birmingham, AL USA

## Abstract

The *N*-methyl-d-aspartate receptor hypofunction is one of the most prevalent models of schizophrenia. For example, healthy subjects treated with uncompetitive *N*-methyl-d-aspartate receptor antagonists elicit positive, negative, and cognitive-like symptoms of schizophrenia. Patients with anti-*N*-methyl-d-aspartate receptor encephalitis, which is likely caused by autoantibody-mediated down-regulation of cell surface *N*-methyl-d-aspartate receptors, often experience psychiatric symptoms similar to schizophrenia initially. However, where and when *N*-methyl-d-aspartate receptor hypofunction occurs in the brain of schizophrenic patients is poorly understood. Here we review the findings from *N*-methyl-d-aspartate receptor antagonist and autoantibody models, postmortem studies on *N*-methyl-d-aspartate receptor subunits, as well as the global and cell-type-specific knockout mouse models of subunit GluN1. We compare various conditional GluN1 knockout mouse strains, focusing on the onset of *N*-methyl-d-aspartate receptor deletion and on the cortical cell-types. Based on these results, we hypothesize that *N*-methyl-d-aspartate receptor hypofunction initially occurs in cortical GABAergic neurons during early postnatal development. The resulting GABA neuron maturation deficit may cause reduction of intrinsic excitability and GABA release, leading to disinhibition of pyramidal neurons. The cortical disinhibition in turn could elicit glutamate spillover and subsequent homeostatic down regulation of *N*-methyl-d-aspartate receptor function in pyramidal neurons in prodromal stage. These two temporally-distinct *N*-methyl-d-aspartate receptor hypofunctions may be complimentary, as neither alone may not be able to fully explain the entire schizophrenia pathophysiology. Potential underlying mechanisms for *N*-methyl-d-aspartate receptor hypofunction in cortical GABA neurons are also discussed, based on studies of naturally-occurring *N*-methyl-d-aspartate receptor antagonists, neuregulin/ErbB4 signaling pathway, and theoretical analysis of excitatory/inhibitory balance.

## Introduction

Schizophrenia is a devastating, chronic psychiatric disorder that is marked by complex and heterogeneous symptoms, such as positive (*e.g.* hallucinations and delusions), negative (*e.g.* anhedonia and social withdrawal), and cognitive symptom (*i.e.* difficulty in attention, memory and executive functions). Much about the etiology and pathophysiology of schizophrenia remains unknown. The dominant hypothesis has implicated hyperactivity of the dopaminergic system.^1^ However, antipsychotics, which mainly block dopamine D2 receptors, show poor efficacy in alleviating the negative and cognitive symptoms. On the other hand, the *N*-methyl-D-aspartate receptor (NMDAR) hypofunction hypothesis has been particularly successful at explaining all of these symptoms associated with schizophrenia.^2^ The uncompetitive or open-channel NMDAR antagonists, such as low-dose of phencyclidine (PCP) and ketamine, induce a psychotic state that resembles schizophrenia more closely than dopamine agonists, because they elicit positive symptoms as well as negative symptoms and cognitive deficits.^3^ Pharmacological intervention with NMDAR antagonists appears to model much of the phenomenology of schizophrenia. However, it remains largely unknown regarding what mechanisms or molecules elicit NMDAR hypofunction that potentially leads to schizophrenia in humans. In order to probe for such mechanisms/molecules, it is important to identify the spatial and temporal boundary condition for NMDAR hypofunction in schizophrenia. In other words, we need to determine where and when functional down-regulation of NMDARs occurs endogenously. Research on uncompetitive NMDAR antagonist-induced psychosis and on anti-NMDAR encephalitis, which often causes schizophrenia-like psychosis, has provided some important clues. However, a more straight-forward approach has been to analyze genetically-engineered animal models with cell-type selective ablation of NMDARs. In this review, several significant findings stemming from these clinical cases and preclinical models are discussed that are relevant for understanding the spatial and temporal boundaries of NMDAR hypofunction.

## Drug model supporting NMDAR hypofunction

Both PCP^4^ and ketamine^5^ exacerbate positive and negative symptoms in pre-existing schizophrenia, suggesting a shared mechanism with schizophrenia pathophysiology. Indeed, both NMDAR antagonists lead to increase the power of cortical gamma oscillations^6^ and in functional connectivity.^7^ The antagonists also induce disturbance in the GABAergic system such as a loss of glutamic acid decarboxylase-67 (GAD67) and parvalbumin (PV), which are consistently reported in postmortem brains of schizophrenia.^8^ Ketamine also increases the glutamate levels in prefrontal cortex or hippocampus in human ^1^H-MRS studies,^9^ which is consistent with the elevated glutamate observed in schizophrenia.^10^ Likewise, NMDAR hypofunction models based on the actions of PCP and ketamine have become increasingly well established, as a major tenet of glutamatergic dysfunction theory of schizophrenia.^2,3^ However, potential mechanisms by which PCP and ketamine lead to these changes have been debated.

Acute systemic treatment with MK-801 in rats has been shown to preferentially block NMDARs on the medial prefrontal cortex (mPFC) GABAergic interneurons, leading to a reduction of GABAergic network function and a subsequent increase in pyramidal cell firing due to disinhibition.^11,12^ Curiously, a local infusion of PCP or MK-801 directly into mPFC does not evoke the excitatory responses of the local pyramidal neurons in awake rats.^13–15^ In contrast, local MK-801 infusion to hippocampal CA1 in adult rats reproduced the disinhibitory effect of systemic MK-801 on the mPFC pyramidal neuron firings. Therefore, Jodo suggested^16^ that MK801-induced excitability of mPFC pyramidal neurons likely results from the activation of excitatory inputs in the ventral part of the hippocampus, which is known to send a dense monosynaptic glutamatergic projection to the mPFC superficial layer.

It is still unclear how locally applied MK-801 results in differential effects on the pyramidal neuron activities of the mPFC and hippocampus. One intriguing possibility is that mPFC pyramidal neurons in vivo are more susceptible to the MK-801 blockade, presumably due to their GluN2B-mediated persistent activity.^17^ In vivo intra-PFC iontophoresis of MK-801 into the primate dorsolateral PFC resulted in a marked decrease in the task-related firing of pyramidal cells,^18^ suggesting that NMDARs are involved in up-state or burst firing in mPFC. In contrast, NMDARs in the hippocampal pyramidal cells are crucial for synaptic plasticity induction,^19^ but they show little effect on the burst firing per se. Thus, the spatial tunings, but not the firing rates, of place cell activity are compromised by NMDAR antagonists and by genetic deletion of NMDAR obligatory subunit GluN1 (NR1). Moreover, it has been reported that over 60 % of the hippocampal complex-spike cells are silent during the exploration of spatial environment and do not display place cell activity.^20^ It is plausible that these “silent” hippocampal principal cells are less sensitive to uncompetitive NMDAR antagonists.

Is there any evidence that NMDAR antagonist directly acts on the NMDARs on hippocampal PV neurons? A recent report has revealed a selective localization of synaptic NMDARs at the feed-back synapses from the recurrent collaterals, but not at the feed-forward synapses from Schaeffer-collateral inputs, of CA1 pyramidal cells^21^ (but see ref. 22). Importantly, the feed-back synaptic input to PV-containing neurons appears to be crucial for their spike timing precision, thereby regulating the emergence of theta/gamma oscillations. Indeed, PCP treatment disrupts type I (atropine-resistant) theta oscillations and increases gamma power in area CA1 in vivo.^23^ GluN1 ablation in PV neurons also resulted in exactly the same phenotypes; a disruption of type I theta oscillations and an increase in spontaneous gamma oscillations in the hippocampus.^24^ These results strongly suggest that PCP acts at NMDARs of the feed-back synapses on PV neurons in adulthood.

Another recent study showed that low concentrations of the NMDAR antagonists PCP and memantine preferentially inhibit tonically-active extrasynaptic NMDARs on the CA1 interneurons containing GluN2D in adult mice.^25^ Interestingly, tonic NMDARs have a more prominent contribution to the intrinsic excitation in GABA neurons than in pyramidal neurons, which is consistent with the excessive disinhibition by NMDAR antagonists in the hippocampus.^26^

It is noted that expression of GluN2D-containing NMDARs is largely confined to cortical and hippocampal GABA neurons, mainly PV-containing.^27^ Also, *ex vivo* data show that GluN2D-containing NMDARs are less-sensitive to Mg^2+^ blockade.^28^ A series of studies by Ikeda and colleagues demonstrated that GluN2D knockout mice are insensitive to PCP or ketamine treatment.^29–31^ For example, PCP increases locomotor activity and extracellular dopamine levels in the striatum and prefrontal cortex, but this increase is not found in GluN2D knockout mice,^29^ suggesting that GluN2D-containing NMDARs are responsible for some of the effects of PCP. Similarly, ketamine-induced baseline gamma power increases are also abolished in GluN2D knockout mice.^32^ These results may suggest that NMDAR antagonists are effective preferentially at GluN2D-containing GABA neurons, leading to schizophrenia-like phenotypes in mice. Taken together, acute NMDAR antagonist-induced psychosis in adulthood appears to be mediated, at least in part, by the GluN2D-containing NMDARs in the hippocampal GABA neurons including PV neurons.

However, repetitive or subchronic treatment of NMDAR antagonists in adulthood may produce more robust phenotypes than those seen following acute treatment. For example, acute administration of NMDAR antagonists increases dopamine level in mPFC, while their long-term treatment results in the reduction of dopamine release in the prefrontal cortex in rats and monkeys.^33^ Since amphetamine-induced dopamine release in prefrontal cortex appears to be compromised in patients with schizophrenia,^34^ chronic treatments may be a better model the dopamine phenotype in prefrontal cortex. Intensive research to identify changes in the brain following repetitive administration of NMDAR antagonists has been reviewed elsewhere.^35–37^

## Autoantibody model supporting NMDAR hypofunction

Compelling clinical evidence supporting the NMDAR hypofunction theory of schizophrenia also comes from studying anti-NMDAR encephalitis. Anti-NMDAR encephalitis is recently described as one of most common synaptic autoimmune disorders. Clinical expression of this disease consists of a variable presentation of psychiatric symptoms such as hallucinations, delusions, mania, catatonia, and insomnia days after the prodromal phase.^38^ About 65% of adults first present with psychiatric symptoms and the majority are initially assessed by the psychiatric services.^39^ IgG antibodies targeting the extracellular domain of the GluN1 subunit of the NMDAR are likely to be the main pathogenesis of the disease.^40^ NMDAR downregulation seems to be due to the reduction of surface NMDARs resulting from antibody-mediated crosslinking of NMDARs leading to internalization of the receptors. Receptor internalization occurs at the same degree in both excitatory and inhibitory neurons, reaching plateau 12 h after auto-antibody treatment in cultured hippocampal neurons.^41^ Consequently, NMDAR-mediated mini-EPSC amplitudes in the pyramidal neurons are significantly reduced 24 h after the antibody added to the cultured cells, while NMDA component in the GABA neurons has not been tested. Because the antibody does not inhibit the NMDA currents, NMDAR hypofunction is likely a result of lower expression of surface receptors, but not due to the functional channel blocking currents.^41^ Therefore, initial presentation of psychiatric symptoms could be associated with the cell-types in which NMDARs are first robustly internalized. Quantitative immunogold electron microscopic study in rat hippocampus showed that GluN1 density is highest in pyramidal cell spines and lowest in dendrites of PV neurons in *st. radiatum*.^42^ Antibody-mediated NMDAR internalization at the PV-positive GABA neurons, which depletes cell-surface NMDARs quickly, may be linked to the initial psychiatric symptoms. When the cerebrospinal fluid (CSF) antibody titers get higher, the pyramidal neurons' NMDARs may also be depleted from the cell surface leading to additional symptoms.^43^ Indeed, CSF antibody titers have been shown to be related to the illness severity of various encephalitic symptoms.^44^ It may be worth assessing whether NMDAR internalization at PV neurons is associated with the psychiatric symptoms of this disorder. Notably, the prevalence of anti-NMDAR autoantibodies in populations with primary schizophrenia appears to be low.^43^ However, the discovery of NMDAR autoantibodies associated with acute psychosis strongly reinforces the NMDAR hypofunction model of schizophrenia as a biological basis for mental illness.

## Developmental aspect of NMDAR hypofunction

Although drug- and autoantibody-mediated NMDAR hypofunction models, which occur in adulthood, show phenomenological validity, pathophysiology of schizophrenia itself remains unexplained. Schizophrenia is a neurodevelopmental disorder and insults to the developing brain can contribute significantly to the manifestation of the disorder in young adulthood.^45^ Therefore, the above models are unlikely to be able to replicate the neurobiological changes that occur over time in the schizophrenic brain. Instead, accumulating evidence suggests that transient NMDAR blockade in perinatal period confers long-lasting brain and behavior change. For example, maternal abuse of PCP during pregnancy increases the risk of the fetus developing schizophrenia later in life.^46,47^ Similarly, ketamine anesthesia during the first week of life also results in long-lasting cognitive deficits in rhesus monkeys.^48^ And a number of studies in rodents have already demonstrated that NMDAR blockade in early postnatal development resulted in the behavioral, neurochemical, and electrophysiological changes, some of which recapitulate the human schizophrenia phenotypes (reviewed by 49,36,50). For example, NMDAR antagonist exposure in postnatal day (PND) 7–11 leads to behavioral abnormalities including learning and memory impairments, hyperlocomotion, impaired sensorimotor gating. The same transient treatments also cause persistent impairments to the function of cortical and hippocampal GABAergic neurons, in particular PV-positive fast-spiking neurons.^51–58^

Robust effects of NMDAR antagonists during early postnatal period are explained by crucial role of NMDARs in brain development.^59,49^ During this period, NMDAR activation is essential for neuronal survival, differentiation, migration and synaptogenesis.^60^ For example, at PND 2 in rodents, many immature neurons positive for NMDARs are still migrating in the cortex, and MK-801 acts as a caspase-3 activator, thereby inducing massive apoptosis in those neurons.^61^ By the first 2 weeks of age, peak expression of NMDARs occur in rats,^62^ and the NMDA currents in immature neurons are larger with longer duration that adult neurons,^63^ due to high expression of GluN2B/2D. NMDA currents are also robust in fast-spiking neurons of mPFC between PND 15–28^64^ and of hippocampus between PND 3–8.^22^ Then, during adolescence synaptic NMDA inputs to intrinsically mature fast-spiking neurons became smaller in mPFC (after PND 28;^64^ 6 weeks of age^65^) and in hippocampal CA1 in juvenile period (PND 14–21), at least partly due to the GluN2B-GluN2A switch.^22^ Interestingly, GluN1 mRNAs are detected after adolescence^66^ and tonic (extra-synaptic) NMDA currents largely remain until adolescence in PV neurons^67^ or even in adulthood.^25^ Importantly, spike timing of cortical fast-spiking neurons and the maturation of their intrinsic properties, which is normally completed by PND 21,^68,69^ are impaired in mice treated with MK-801 at PND 6–8.^70^ Disturbance of GluN2B-GluN2A switch in fast-spiking neurons also occurs following transient treatment at PND 7–11 with PCP,^71^ ketamine^57^ or MK-801^70^, resulting in the increased GluN2B component in NMDA currents in PV neurons. Overall, uncompetitive NMDAR antagonists disrupt the maturation of intrinsic excitability of fast-spiking neurons during this period,^70^ which could have an impact on the circuitry maturation and on the excitatory/inhibitory (E/I) balance.

## Genetically-engineered global NMDAR hypofunction

**Fig. 1:**
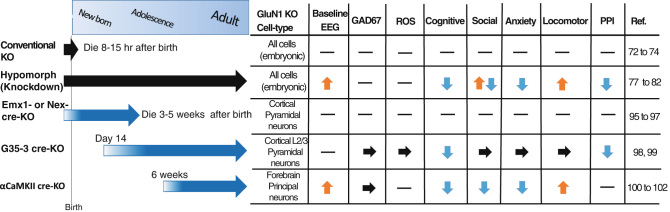
Summary of schizophrenia-related phenotypes in various GluN1 mutant mice. GluN1 knockdown regardless of cell-types ("hypomorph mouse") presents several aspects of schizophrenia-related signs. ﻿GluN1 deletion﻿﻿in glutamatergic neurons (Emx1﻿-cre KO and Nex-cre KO)during development both result in the phenotypes too severe to consider as schizophrenia-related signs. GluN1 deletion in forebrain principal neurons in adulthood (G35-3 cre-KO and αCaMKII cre-KO) appears to be sufficient to cause some neuropsychiatric signs, including E/I balance increase and cognitive dysfunction; however, insufficient to induce abnormalities of local GABA neurons seen in schizophrenia pathophysiology. Mouse strains with *black* arrows (top two strains) received genetic manipulation targeted to all the cells throughout the development. The manipulation in the mouse with *blue* arrows (bottom three strains) was largely restricted to the particular cell-types of forebrain principal neurons. *Thick arrows* in the *left panel* show the period of knockout occurring in the designated KO cell-type in the cortex. Hyphen denotes no data in the right Table. *ROS* reactive oxygen species. *PPI*, prepulse inhibition of startle reflex

Having no off-target effects, genetically-engineered mice have been excellent tools to assess the spatial and temporal boundary conditions of NMDAR hypofunction (Fig. 1). Though complete genetic ablation of GluN1, a vital subunit of NMDARs, may lead to an overly pronounced phenotype that does not share the genetic basis of schizophrenia, it may be a useful tool to model the effect of NMDAR antagonists as it leads to a functional blockade of the receptor. Conventional GluN1 knockout mice are not viable, dying 8–15 h after the birth.^72^ However, careful analyses revealed severe disruption of somatosensory barrel cortex formation,^73^ which appears more sensitive than that of schizophrenia pathology. Furthermore, corpus callosum development is rather accelerated in both the mutants and GluN1 haploinsufficiency (+/-) mice compared to the wild type mice.^74^ In contrast, the volume of central corpus callosum is known to be significantly decreased in schizophrenia,^75^ with effects more detectable in first-episode than chronic schizophrenia.^76^ In this context, global NMDAR hypofunction in the late gestation period is unlikely to take place in schizophrenia pathogenesis.

Several genetically-engineered mouse models have been generated to overcome the neonatal lethality of GluN1 conventional knockout mice. Among them, a notable model is a GluN1 “hypomorph” mouse line,^77^ which was generated by inserting the neomycin cassette into the GluN1 gene, thereby resulting in the GluN1 protein expression approximately 10% of the normal level that survived to adulthood (Fig. 1). Subsequent studies found decreased PPI, sociability and mating behavior as well as impaired olfaction and increased locomotor activity. Based on these findings, GluN1 hypomorph mutant strain has been described as a transgenic model of schizophrenia.^78^ However, behavioral anomalies began to appear before the weaning,^79^ and the adult mutants displayed robust impairments in short and long-term memory, on both spatial and non-spatial tasks, which made it difficult to dissociate a mnemonic phenotype from a more general performance deficit.^80^ Siegel and colleagues also investigated sensory event-related potentials (ERPs) of this strain and found a significant sensory disinhibition; but its ERP pattern was atypical for patients with schizophrenia,^81^ rather closely resembles what is seen in autism.^82^ These findings indicate that GluN1 hypomorph mice may show a more global impairment rather than that which belongs to any specific disease. Hence, it is expected that region-specific or cell type-specific deletions of GluN1 subunit may uncover more precise contribution of NMDARs to the emergence of the schizophrenia-like phenotypes.

## NMDAR hypofunction targeted to cortical excitatory neurons

Alteration of NMDAR subunit GluN1 mRNA has been often reported to decrease by 15–30% in the post-mortem brains with schizophrenia patients^83–89^, while some reports showed no change^90^ or an increase.^91^ Although the findings are conflicting, one may speculate that GluN1 expression may be down-regulated in the cortex and hippocampus of patients with schizophrenia (see review by 92,93), which could lend support for NMDAR hypofunction in the cortical and hippocampal pyramidal neurons. Additionally, even if the receptor levels were not lowered, postsynaptic NMDA signaling, such as Src kinase, may still be down-regulated in schizophrenia.^94^ However, it is unclear regarding which developmental stage such NMDAR hypofunction takes place in pyramidal neurons in patients with schizophrenia. In animal studies, several genetically-engineered mouse strains in which GluN1 ablation was targeted to cortical and hippocampal glutamatergic excitatory neurons have been analyzed. Two embryonic GluN1 knockout mouse strains were generated, in both of which the NMDAR deletion is selective to cortical and hippocampal glutamatergic neurons^95,96^ (Fig. 1). Both knockout mice showed severe growth retardation and most died within 3–5 weeks after the birth,^96,97^ suggesting that embryonic NMDAR knockout in the glutamatergic neurons results in the phenotypes which are too severe to be considered as schizophrenia-related signs.

To prevent the premature lethality, we generated and analyzed a GluN1 knockout strain^98^ (Fig. 1), in which postnatal GluN1 deletion is largely confined to the excitatory neurons in layer II/III of mPFC and sensory cortical areas, but not in the hippocampus or any GABA neurons.^99^ These mutant mice showed cognitive deficits in PPI and object-based attention tasks, suggesting that NMDAR hypofunction in cortical excitatory neurons could contribute to cognitive dysfunction. However, no other phenotypes were detectable in the tests of locomotion, social interaction, saccharine preference, and anxiety. We also found no alteration in mini-EPSCs and mini-IPSCs in the layer II/III pyramidal cells, no deficits in Gad67 expression, or no sign of reactive oxygen species (ROS) increase in the cortical area that received GluN1 ablation in pyramidal neurons. Thus, the cortical layer II/III pyramidal neuron knockout of GluN1 is unlikely to elicit GABAergic dysfunction and oxidative stress response.

Recently, the impact of GluN1 deletion in forebrain principal neurons on schizophrenia-related phenotypes was investigated using αCaMKII promoter-driven GluN1 knockout^100^ (Fig. 1). The mutant strain was generated by the same intercross between the Cre line and the floxed-GluN1 line as previously reported.^101^ GluN1 knockout begins in area CA1 at 6 weeks of age and spreads out into other telencephalic regions including area CA3 and entorhinal cortex by 4 months of age, but not in local GABA neurons.^102,100^ Physiological analysis in 3–5 month-old slices revealed an increase in CA1 pyramidal neuron excitability with spontaneous EPSC frequency increase. This could be attributable to G protein-regulated inward-rectifier potassium channel 2 reduction in the GluN1-deleted neurons, as suggested in the study. Alternatively, a diminished feed-forward inhibition from GluN1-deleted CA3 and/or entorhinal pyramidal neurons may occur, since selective GluN1 ablation in CA3 pyramidal neurons results in CA1 disinhibition, leading to kainate-induced excitotoxicity.^103^ Moreover, the network disinhibition may cause the increase in spontaneous neuronal activity or “noise”. Consistently, αCaMKII -GluN1 mutant mice showed a broadband LFP power increase and an impaired evoked power regardless of the frequency at area CA3 in the auditory paired-click paradigm. Combined with behavioral data showing impairment in spatial working memory and reduced nest building and social activity, it was proposed that NMDAR hypofunction in pyramidal neurons is sufficient to cause schizophrenia-related changes.^100^ In this study, however, NMDAR knockout in pyramidal neurons did not recapitulate a dysfunction of local GABAergic neurons, such as a reduction of GAD67 and PV mRNAs, which is consistently reported in the postmortem brain of schizophrenia.^8^ Together with our previous report,^99^ pyramidal neuron-NMDAR hypofunction in adulthood appears to cause neuropsychiatric signs, including E/I balance increase and cognitive dysfunction; however, insufficient to induce abnormalities of local GABA neurons seen in schizophrenia pathophysiology.

## NMDAR hypofunction targeted to cortical PV neurons

Despite the abundance of NMDAR expression in the cortical and hippocampal pyramidal neurons, systemic administration of uncompetitive NMDAR antagonists (PCP, ketamine and MK-801) in rodents has been consistently demonstrated to increase E/I balance and enhance disinhibition (reviewed by 104–108). Importantly, in vivo effects of MK-801 first appeared as reduced activity of GABA neurons, followed by increased activity of pyramidal neurons in mPFC.^12^ It has also been shown ex vivo that hippocampal GABAergic interneurons are disproportionally more sensitive to NMDAR antagonists than pyramidal neurons.^26^ Although the reason why uncompetitive NMDAR antagonists preferentially impact GABA neurons is not completely understood, at least two mechanisms are suggested. First, local GABA neurons, in particular, fast-spiking neurons containing PV, are known to fire action potentials at much higher frequency than pyramidal neurons and the interneurons positive for somatostatin, vasoactive-intestinal-polypeptide or neuropeptide Y.^109^ Therefore, the channel pore Mg^2+^ occlusion may be relieved by constant depolarization. Second, ketamine is reported to preferentially inhibit GluN2C- and GluN2D-containing NMDARs in artificial CSF with physiological Mg^2+^ concentration, presumably due to their reduced affinity to Mg^2+^.^28^ Importantly, GluN2D-containing NMDAs are selectively distributed in PV-positive and/or somatostatin-positive interneurons of the cortex and hippocampus in adulthood.^110,111,27^ Therefore, a specific deficit in NMDAR signaling in fast-spiking PV neurons may explain certain aspects of NMDAR hypofunction-mediated schizophrenia-related phenotypes.

**Fig. 2:**
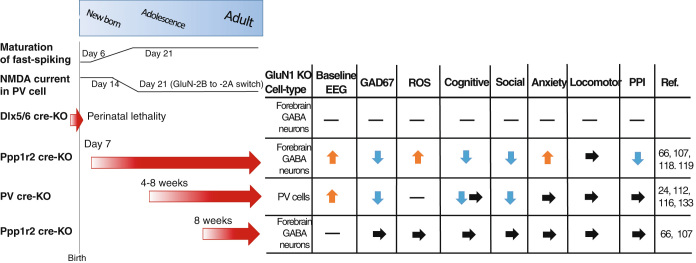
Summary of schizophrenia-related phenotypes in various GluN1 mutant mice, in which GluN1 knockout is targeted to GABA neurons. GluN1 deletion during the embryonic stage in migrating forebrain Dlx5/6-positive GABAergic cells lead to lethality(Dlx5/6 cre KO). In contrast, GluN1 deletion after postnatal 4 weeks in PV neurons (PV-cre KO) or after adolescence in Ppp1r2-positive GABA neurons (bottom strain) produce less phenotypes compared to the mutant mice that received GluN1 deletion in GABA neurons in early postnatal period (Ppp1r2 cre-KO starting from PND 7). Thus, it appears that early postnatal period, but not during the embryonic stage or after adolescence, is a critical period of GABAergic NMDAR hypofunction leading to schizophrenia. *arrow* shows the period of knockout occurring in the designated KO cell-type in the cortex. The level of intrinsic property maturation of neocortical fast-spiking neurons largely based on Refs. 68,69. Relative change in synaptic evoked NMDA component estimated from the data in Ref. 22 for hippocampal PV neurons and Ref. 65 for mPFC PV neurons. Hyphen denotes no data. The data of Dlx5/6 cre-KO mice is unpublished. *ROS* reactive oxygen species. *PPI*, prepulse inhibition of startle reflex

To test this hypothesis, conditional deletion of the GluN1 subunit selectively in PV-positive cells was achieved by two groups, using the same PV-cre line, which was produced by Silvia Arbor’s lab, with a floxed-GluN1 line distinct each other,^24,112^ (Fig. 2). Both PV-cre/GluN1 KO mutants demonstrated cortical and hippocampal disinhibition and the baseline gamma power increase. More importantly, induction of gamma oscillation driven by PV neuron-selective optogenetic activation was impaired in the mutants,^112^ which strongly supports the idea that PV neuron is necessary for generation of gamma-band oscillations.^113,114^ Notably, baseline gamma power increase and sensory-evoked gamma oscillation deficits are consistently demonstrated by NMDAR antagonist exposure to animals^6^ and in EEG/MEG studies in patient with schizophrenia.^115^ Furthermore, in vivo MK-801 action on PV neurons was supported by the finding that MK-801–induced stereotypies are undetectable in the PV-cre/GluN1 KO mutant mice.^112^ The mutants also showed selective impairments in habituation, short-term memory, sociability and vocalizations, although they displayed normal PPI for acoustic startle reflex and nest building behavior^24,112,116^ (Nakazawa, unpublished). One probable reason for less behavioral deficits compared to those observed in the Ppp1r2cre/GluN1 KO mutants (see below for details) may be related to the onset of GluN1 knockout in PV neurons. Immunocytochemistry data suggest that the onset of GluN1 knockout is within 4–8 weeks of age for both mutants,^112,116^ which is much later than the maturation period of the fast-spiking property of PV neurons (PND 6–21.^68,69^). Actually, the evoked NMDA components on the glutamatergic synapses in PV neurons in the mPFC^64,65^ and hippocampus^22^ seem to be diminished by PND 21–28. Alternatively, additional NMDAR deletion in other GABA neurons, like Reelin-positive GABA neurons, may also be important for the robust behavioral phenotypes, because approximately 30% of Reelin-positive GABA neurons are also Cre-targeted in the cortex and hippocampus of the Ppp1r2-cre mouse strain.^66^

## NMDAR hypofunction targeted to GABA neurons in development

We predicted that NMDAR knockout in GABA neurons during a much earlier developmental period may be necessary to produce more robust phenotypes. To target the GluN1 ablation into GABA neurons in development, we crossed the floxed-GluN1 mice to a Dlx5/6-cre line, in which Cre recombination occurs in migrating forebrain GABA neurons of the embryonic brain.^117^ However, we found that no homozygously-floxed mutant mice survived, suggesting that NMDAR function in migrating GABA neurons is fundamental for brain development and survival, but beyond the schizophrenia pathophysiology (Nakazawa, data not shown).

To target the GluN1 ablation into early postnatal development of GABA neurons, we crossed the floxed-GluN1 mice to a Ppp1r2-cre line, in which Cre expression is detected by PND 7 in 40–50% of cortical and hippocampal GABA neurons,^66,107^ (Fig. 2). In the somatosensory (S1) cortex, about 70% of cre-targeted neurons are PV containing, and the rest includes Reelin-positive neurons, but negligible in the pyramidal neurons. Since cre recombination occurs in over 80, 75, and 90% of PV neurons in the neocortex, mPFC and hippocampus, respectively, it is expected to confer efficient genetic manipulation in PV neuron cell-type. Genetic ablation of GluN1 in Ppp1r2-positive neurons^66^ results in a variety of behavioral abnormalities, such deficits in as spatial and social short-term memory, nest building, and PPI. The mutants also displayed increased anxiety-like behavior in the elevated plus maze and open field, and novelty-induced hyper locomotion. Interestingly, most behavioral abnormalities appeared after young adulthood (8 weeks old), some of which were exacerbated by the social isolation stress.^66,118^ Furthermore, in vivo awake LFP/unit recordings showed not only an increased pyramidal cell firing and their action-potential spike synchrony deficits in S1 cortex^66^ but also revealed a baseline broadband LFP power increase and impaired tone-evoked gamma power amplitude and phase locking in primary auditory cortex.^119^ These physiological phenotypes are shared with PV-cre/GluN1 KO mutants, suggesting that these gamma synchrony deficits are attributed to GluN1 deletion in PV neurons. Interestingly, auditory tone stimulation also evoked prolonged activation of mutants’ auditory deep layer pyramidal neurons in a 5-HT2A receptor-dependent manner (Nakao *et al.*, unpublished). Moreover, hypo-dopaminergic state in mPFC and hyperdopaminergic state in the striatum appears to co-occur in this mutant brain (Nakao *et al.*, unpublished). Therefore, both serotonin and dopamine dysregulation, which are reminiscent of schizophrenia, appear to be a downstream event of developmental NMDAR hypofunction in GABA neurons. Interestingly, when GluN1 was deleted in the Ppp1r2 neurons after 8 weeks of age (because the recombination onset was delayed in another floxed-GluN1 line used), we found no behavioral or dopaminergic impairments. Therefore, early postnatal period, after gestation period but before adolescence, appeared to be a critical period during which NMDAR hypofunction can induce the emergence of schizophrenia-like phenotypes (Fig. 2). A drawback with using the Ppp1r2cre line is that the knockout occurs not only in local PV neurons but also in other cortical GABA neurons, including Reelin-positive interneurons. Cre expression is also detected in a majority of neurons of tenia tecta and lateral septum, and a subset of neurons in superior colliculus superficial layers. Thus, behavioral deficits of the mutants could be attributed to abnormalities of such subcortical GABA neurons. Interestingly, Cre expression is detected only 5% GABA neurons of thalamic reticular nucleus in Ppp1r2cre line, which are mostly positive for PV. Further studies are warranted to determine if the early postnatal PV neurons are the locus of NMDAR hypofunction.

## Additional findings supporting NMDAR hypofunction in cortical GABA neurons

The evidence for NMDAR hypofunction in GABA neurons is scarce. However, a recent postmortem meta-analysis quantifying NMDAR subunit expression in the prefrontal cortex of people with schizophrenia found a reduction of GluN2C subunit mRNA in all three studies examined.^93^ Expression of cortical GluN2C subunit is confined to subsets of interneurons in the cortex. Therefore, a GluN2C expression deficit could account for NMDAR hypofunction in GABA neurons in schizophrenia. Another postmortem study demonstrated a lower density of excitatory synapses on PV-positive interneurons in PFC of patients with schizophrenia, while the glutamate receptor subtypes were unidentified.^120^ This could be due to a deficit in excitatory synapse formation on PV neurons, or due to reduced synaptic input from excitatory neurons.

It then must be considered whether there are any potential mechanisms that cause NMDAR hypofunction in GABA neurons during the perinatal stage. A series of endogenous NMDAR antagonist studies allows us to speculate that these antagonists may preferentially act on the NMDARs in the GABA neurons. For example, kynurenic acid (KYNA) is a naturally occurring antagonist of NMDAR at the glycine binding site.^121^ A KYNA synthesizing enzyme, kynurenine aminotransferase II (KAT II) is known to be responsible for brain KYNA synthesis. Interestingly, a recent immunocytochemical study in mice demonstrated that KAT II is expressed predominantly in GABAergic neurons as well as in astrocytes, but much less in the pyramidal cells in the cortex and hippocampus, raising a possibility that locally-secreted KYNAs preferentially inhibits NMDARs in GABA neurons.^122^ Notably, the brain levels of KYNA are elevated in schizophrenia and by the neuroinflammatory stress, which exists in schizophrenia as well.^121^ Mounting preclinical evidence also suggest that KAT II-selective inhibitors produce a pro-cognitive effect in schizophrenia-relevant behavioral assays.^123^ Another study also reported that the effect of KYNA-induced NMDAR blockade on the neuronal differentiation *in vitro* is more prominent on GABAergic neuron lineage compared to glutamatergic neurons, although the underlying mechanisms of the preferential action to GABA neurons is unclear.^124^

Another endogenous NMDAR antagonist which is known to bind to GABAergic NMDARs is a class of sulfated neuroactive steroids, including pregnanolone sulfate (3α5βS; 20-Oxo-5β-pregnan-3α-yl-sulfate) and pregnenolone sulfate (20-Oxo-pregn-5-en-3α-yl sulfate). These compounds are known to act as a use-dependent allosteric NMDAR antagonist, while they also modulate the GABA_A_ receptor.^125^ The levels of the neurosteroids increase towards parturition during pregnancy^126^ and by the acute stressor. Interestingly, it has been reported that these sulfated neurosteroids preferentially bind to tonic NMDARs containing GluN2C and/or GluN2D.^127^ Unexpectedly, however, systemic infusion of the neurosteroid did not elicit psychotomimetic-like behavior in rats and it rather ameliorated MK-801-induced behavioral deficits.^128^ Therefore, further study is warranted to determine the action of these steroids and to what extent they inhibit tonic NMDARs in GABA neurons.

If these events would actually occur in the schizophrenia brain, NMDAR hypofunction in GABA neurons might be the secondary event to environmental insults. However, certain genetic mechanisms may also explain GABAergic NMDAR hypofunction. Buonnano’s team elegantly showed a selective internalization of NMDARs from the cell surface of the cortical ErbB4-expressing GABA neurons, but not in the pyramidal neurons in hippocampal cell culture prepared from E19 rat pups.^129^ ErbB4 and its ligand neuregulin (NRG)-2 are predominantly co-expressed in the PV-containing GABA neurons, but not in the pyramidal neurons in the cortex. Dysregulated ErbB4 splicing is known to associate with PV neurons in schizophrenia.^130^ They demonstrated that NMDARs in GABA neurons are required to release NRG2, which activate its receptor ErbB4 on the same neuron as an autocrine signaling. This activation promotes the association of ErbB4 to GluN2B-contaning NMDARs, and triggers rapid internalization of surface NMDARs, but not AMPA receptors, in the GABA neurons. Therefore, NMDAR seemed to be both an effector and a target of NRG2/ErbB4 signaling in the cortical interneurons. Consistently, it was previously shown that NRG-induced suppression of NMDAR activation is more pronounced in schizophrenia patients than in controls.^131^ These results suggest a plausible cellular mechanism by which NMDARs can be selectively internalized in the cortical PV neurons, but not in pyramidal neurons. Together with the GluN2D knockout studies, these findings may offer a novel therapeutic approach for treating schizophrenia by enhancing the NMDAR function selectively in the GABA neurons.

Theoretical findings also offer supportive evidence for a role for NMDAR hypofunction in PV neurons in the development of schizophrenia. A spiking neural network model of spatial working memory, consisting of interconnected excitatory pyramidal cells and inhibitory interneurons in prefrontal cortex, postulated a preferential down-regulation of NMDAR conductance on interneurons, but not simultaneously in both excitatory and inhibitory neurons, in order to increase E/I balance for working memory deficits in schizophrenia.^132^ The simulation showed that elevated E/I balance state, which seems to occur in schizophrenia, could be increased when the strength of NMDAR conductance on interneurons is robustly reduced while excitatory cells’ NMDAR conductance is maintained. In contrast, when NMDAR conductance on excitatory cells is too low (by reduced E/I balance), the circuit simply could not support persistent activity, so that activity pattern is collapsed during the delay period and the memory was lost. When NMDAR conductance at both pyramidal cells and interneurons is proportionally reduced, E/I balance is unaltered as expected. From this network simulation model, it appears that NMDAR hypofunction selectively on GABA neurons modes the elevated E/I balance in the prefrontal cortex seen in schizophrenia, and that NMDAR hypofunction at both pyramidal neurons and interneurons is unlikely from the E/I balance perspective.

## Potential NMDAR hypofunction in other brain cell-types

NMDAR hypofunction on PV-positive cortical and hippocampal fast-spiking neuron appears to be at the core of schizophrenia pathophysiology.^105–108^ However, this hypothesis has recently been challenged by a preclinical finding that systemic administration of MK-801 to the PV-cre/GluN1 KO mice leads to the increased stereotypies, catalepsy, and motor discoordination that are not observed in the absence of MK-801.^133^ These results led the authors to conclude that NMDAR deletion at PV neurons in adulthood predisposes or sensitizes the circuit to trigger the NMDAR hypofunction in other non-PV neurons. Alternatively, since GluN1 deletion in this mutant line is completed by 8 weeks old,^133^ GluN1 deletion in PV neurons may be too late to elicit robust the schizophrenia-like behavior. Certainly, however, there may be several other candidate cell-types for NMDAR hypofunction of schizophrenia. For example, some cortical PV-negative interneurons, such as somatostatin-positive neurons, contain GluN2D-containing NMDAR channels, which are resistant to Mg^2+^ block at the resting membrane potential. GluN2D-containing NMDARs are also expressed in the dopamine neurons which are negative for PV. Selective deletion of NMDARs from dopamine neurons resulted in an impairment in phasic dopamine release and in instrumental learning.^134^

Notably, GluN2C-containing receptors are highly expressed in the reticular nuclei as well as the relay nuclei in thalamus. Delta oscillations in the thalamocortical system are generated through an interaction of the thalamic relay nuclei and GABAergic reticular nuclei, depending on the T-type Ca^2+^ channels.^135,136^ Since the tonic activation of reticular nuclei is NMDAR-dependent, their NMDAR blockade produces hyperpolarization, thereby activating T-type Ca^2+^ channels. These channel activation produces delta frequency bursting in this circuitry, which occurs during sleep. Interestingly, delta oscillation power is abnormally high in schizophrenia during wakefulness and is also induced by NMDAR antagonist treatment. It is predicted that NMDAR hypofunction in the reticular nuclei could result in elevated waking delta oscillations, which may explain cognitive deficits in schizophrenia. Since the reticular nuclei are positive for PV, this idea may be testable in PV-cre/GluN1 KO mutant mice. GluN2C-containing NMDARs in the relay nuclei, such as nucleus reuniens (negative for PV), might also be a candidate cell-type for NMDAR hypofunction.^137^ Cell-type specific deletion of NMDAR subunit in such cell-types would be a powerful approach to explore whether NMDA receptor hypofunction in the thalamic neurons leads to schizophrenic-like phenotypes.

## Unified hypothesis of NMDAR hypofunction

NMDARs are expressed in most of cells in the central nervous system from the embryonic stage. Since conventional GluN1 knockout shows perinatal lethality and GluN1 hypomorph mice display more diverse phenotypes than what is observed in schizophrenia, NMDAR hypofunction leading to schizophrenia should have spatial and temporal boundaries. The results of studies utilizing uncompetitive NMDAR antagonists suggest that NMDAR hypofunction at PV-positive GABA neurons causes GABAergic disinhibition and glutamate increase, which in turn elicits dopamine dysregulation.^105–108^ Since synaptic NMDARs in PV neurons are more highly expressed during development than adulthood, NMDA hypofunction on PV neurons during development may contribute to the disease. Consistently, drug manipulation during the postnatal second week by NMDAR antagonists recapitulates several schizophrenia-like phenotypes. Similarly, forebrain GABA neuron GluN1 KO mice^66,107^ confers several behavioral and electrophysiological phenotypes that seem to mimic aspects of schizophrenia. On the other hand, NMDAR hypofunction in cortical pyramidal neurons is also strongly suggested, mainly because the levels of GluN1 mRNA/protein are reduced in prefrontal cortex of patients with schizophrenia in postmortem tissue studies.^92,93^ How do we reconcile these results each other?

**Fig. 3:**
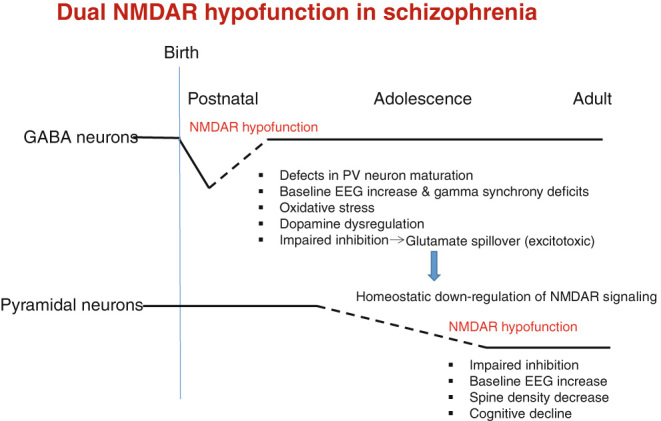
Dual NMDAR hypofunction model for schizophrenia pathophysiology. Accumulating evidence from genetic and pharmacological studies lend support for spatially and temporally dissociated NMDAR hypofunction events in schizophrenia pathophysiology. The first NMDAR hypofunction occurs in cortical GABA neurons, including PV neurons, during early postnatal development, impairing the cortical circuitry maturation and leading to a variety of fast-spiking neuron-associated abnormalities, including gamma band synchrony deficits, oxidative stress increase, dopamine dysregulation, and disinhibition of pyramidal neurons. These abnormalities could contribute to the emergence of positive, negative and cognitive symptoms. Moreover, the E/I balance increase eventually elicits glutamate spillover during the prodromal period or later, which could produce a second NMDAR hypofunction in pyramidal neurons in response to low level but cumulative excitotoxicity. NMDAR hypofunction in pyramidal neurons may be responsible for spine density alteration of cortical pyramidal cells, which could also contribute to cognitive and negative symptoms as the disease progresses. Therefore, both types of NMDAR hypofunction may contribute to the schizophrenia pathophysiology. It is unclear when the first NMDAR hypofunction ends and the second hypofunction starts (shown by *dotted lines*). The cause of the first NMDAR hypofunction remains to be determined (but see text)

Recent clinical studies suggest that elevation in glutamatergic metabolites is present prior to onset of psychosis,^10^ which could be originated from disinhibition by GABAergic NMDAR hypofunction. This glutamatergic spillover could explain the subsequent induction of NMDAR hypofunction in cortical pyramidal neurons, because over-activation of NMDARs by glutamate and glycine elicits a homeostatic internalization of NMDARs from neuronal cell surface.^138,139^ Therefore, we speculate that there are two spatially and temporally dissociated NMDAR hypofunction events that are related to schizophrenia pathophysiology (Fig. 3). The first NMDAR hypofunction occurs in cortical GABA neurons, including PV neurons, in early postnatal development, which would impair the cortical maturation that causes a reduction of intrinsic excitability and impaired GABA release, thereby leading to disinhibition of pyramidal neurons. Subsequently, the second NMDAR hypofunction is in turn elicited in cortical pyramidal neurons in response to glutamate spillover in the prodromal period. Importantly, neither NMDAR hypofunction alone is fully explanatory. For example, schizophrenia-related GABAergic dysregulation, such as reduced expression of GAD67 and PV, is not observed in excitatory neuron GluN1 knockout mice^99,100^ but is consistently reported in the GABAergic GluN1 knockout mice. On the other hand, spine density reduction is induced by GluN1 knockdown in the pyramidal neurons.^96,140,141^ Spatial reference memory in Morris water maze test is intact in PV-cre/GluN1 KO mice^112^ but is severely impaired in αCaMKII promoter-driven GluN1 knockout mice.^101^ It is plausible that cognitive decline observed in a subtype of chronic patients may be explained by NMDAR hypofunction in cortical pyramidal neurons. Further studies are warranted to determine the contribution of each NMDAR hypofunction to the disease pathophysiology. It must be noted that NMDAR hypofunction in other cell-types could occur in a temporally-specific manner in addition to these two events, which are not mutually exclusive.

## Outlook

The developmental NMDAR hypofunction hypothesis in GABA neurons remains highly speculative, because there is no convincing evidence of reduced NMDAR-mediated glutamatergic neurotransmission onto PV neurons in patients with schizophrenia. One way to address this issue is to identify the regulator molecules that could elicit NMDAR channel down-regulation in cortical GABA neurons. The NMDAR complex comprises hundreds of intracellular proteins, and its function is regulated at many levels, including transcription, translation, post-translation, protein trafficking, channel modulation, and cellular activity, as well as possibly being developmentally regulated. Therefore, any combination of genetic and epigenetic mutations could render NMDAR signaling pathways less active, which may allow schizophrenia to develop. Indeed, many NMDAR-associated genes are listed as a schizophrenia risk gene,^142^ such as NRG2/ErbB4 signaling pathway, which causes NMDAR downregulation selectively in PV neurons.^129^ Genome-wide association studies of schizophrenia also uncover genetic polymorphisms in several immune-related genes,^142^ which may affect the levels of proinflammatory cytokines, leading to activation of kynurenine pathway.^143^ The discovery of abundant expression of KYNA synthesizing enzyme in cortical GABA neurons^122^ may allow us to speculate NMDAR hypofunction in GABA neurons induced by the neuroinflammatory stress. The glutathione (GSH) system dysfunction is also seen in schizophrenia.^144^ Since NMDAR is required for GSH biosynthesis, MK-801 treatment at PND 7 in rats was shown to reduce GSH levels, which in turn may trigger redox imbalance and further suppression of NMDA currents as a negative spiral.^145,146^ Notably, PV-positive fast-spiking neurons are highly vulnerable to early life-oxidative stress,^147^ perhaps because fast-spiking neurons have an increased demand for GSH. It remains to be tested whether developmental NMDAR hypofunction occurs on PV neurons by elevated oxidative stress.

Alternatively, it will also be beneficial to examine whether animals’ molecular signs following neonatal NMDAR blockade is observed in the postmortem human brain tissues. Neonatal treatment with uncompetitive NMDAR antagonists disrupts the maturation of the fast-spiking property.^70^ GluN1-deleted fast-spiking neurons in Ppp1r2-cre/GluN1 KO mutant mice also exhibit the maturation deficits of fast-spiking neurons, such as significantly low instantaneous firing rates and high input resistance in juvenile period (Jeevakumar, unpublished). Therefore, it is tempting to speculate that the signature of NMDAR blockade-mediated maturation deficits in PV neurons could be found in human patient tissues. For example, neonatal repeated exposure to PCP,^71^ ketamine,^57^ or MK-801^70^ consistently disturbs the developmental GluN2B-GluN2A switch that occurs in immature PV neurons during PND 14–21.^22^ Consistently, a transcription factor called REST, which facilitates GluN2B-GluN2A switch in neurons, appears to be less functional in schizophrenia.^148^ If the increase of GluN2B subunit mRNA was detected in PV neurons of schizophrenia postmortem brains, in particular, at the earlier life stage, it might suggest NMDAR hypofunction in PV neurons in perinatal period.

In conclusion, multiple strands of evidence from the animal studies suggest that there are two spatially and temporally dissociated NMDAR hypofunction events that are related to schizophrenia pathophysiology (Fig. 3). One is the NMDAR hypofunction in early postnatal development on cortical and hippocampal GABAergic interneurons linked to the primary pathophysiology of schizophrenia. The resulting GABAergic deficit-mediated E/I ratio elevation may produce the glutamate spillover, which could result in the homeostatic down-regulation of NMDAR-signaling pathway in cortical pyramidal neurons in prodromal period. Other cell-types, including the reticular and relay nuclei of the thalamus, may also be responsible for some of the symptoms, and these possibilities are not mutually exclusive. Although technically challenging, it will be important to directly assess the NMDAR hypofunction at the cellular levels in human brains. Recent development of NMDAR-subunit selective allosteric modulators would be promising for potential pharmacological interventions. More coordinated efforts between human and animal studies will be necessary to understand the NMDAR hypofunction leading to schizophrenia and ultimately to reverse the related symptoms.

## References

[1] Howes OD, Kapur S (2009). The dopamine hypothesis of schizophrenia: version III--the final common pathway. Schizophr. Bull..

[2] Coyle JT (2012). NMDA receptor and schizophrenia: a brief history. Schizophr. Bull..

[3] Lodge D, Mercier MS (2015). Ketamine and phencyclidine: the good, the bad and the unexpected. Br. J. Pharmacol..

[4] PCP (Phencyclidine): Historical and Current Perspectives, Edward F. Domino, ed. NPP Books, Ann Arbor, USA (1981).

[5] Lahti AC (1995). Subanesthetic doses of ketamine stimulate psychosis in schizophrenia. Neuropsychopharmacology..

[6] Hunt MJ, Kasicki S (2013). A systematic review of the effects of NMDA receptor antagonists on oscillatory activity recorded in vivo. J. Psychopharmacol..

[7] Driesen NR (2013). Relationship of resting brain hyperconnectivity and schizophrenia-like symptoms produced by the NMDA receptor antagonist ketamine in humans. Mol. Psychiatry.

[8] Gonzalez-Burgos G, Fish KN, Lewis DA (2011). GABA neuron alterations, cortical circuit dysfunction and cognitive deficits in schizophrenia. Neural Plast..

[9] Stone JM (2012). Ketamine effects on brain GABA and glutamate levels with 1H-MRS: relationship to ketamine-induced psychopathology. Mol. Psychiatry.

[10] Merritt K. *et al.* Nature of Glutamate alterations in schizophrenia: a meta-analysis of proton magnetic resonance spectroscopy studies. *JAMA Psychiatry* **73**, 665–674 (2016). 10.1001/jamapsychiatry.2016.0442 27304221

[11] Jackson ME, Homayoun H, Moghaddam B (2004). NMDA receptor hypofunction produces concomitant firing rate potentiation and burst activity reduction in the prefrontal cortex. Proc. Natl. Acad. Sci. USA.

[12] Homayoun H, Moghaddam B (2007). NMDA receptor hypofunction produces opposite effects on prefrontal cortex interneurons and pyramidal neurons. J. Neurosci..

[13] Gratton A, Hoffer BJ, Freedman R (1987). Electrophysiological effects of phencyclidine in the medial prefrontal cortex of the rat. Neuropharmacology.

[14] Suzuki Y (2002). Acute administration of phencyclidine induces tonic activation of medial prefrontal cortex neurons in freely moving rats. Neuroscience.

[15] Jodo E (2005). Activation of medial prefrontal cortex by phencyclidine is mediated via a hippocampo-prefrontal pathway. Cereb. Cortex.

[16] Jodo E (2013). The role of the hippocampo-prefrontal cortex system in phencyclidine-induced psychosis: a model for schizophrenia. J. Physiol. Paris.

[17] Monaco SA, Gulchina Y, Gao WJ (2015). NR2B subunit in the prefrontal cortex: A double-edged sword for working memory function and psychiatric disorders. Neurosci. Biobehav. Rev..

[18] Wang M (2013). NMDA receptors subserve persistent neuronal firing during working memory in dorsolateral prefrontal cortex. Neuron.

[19] Nakazawa K (2004). NMDA receptors, place cells and hippocampal spatial memory. Nat. Rev. Neurosci..

[20] Thompson LT, Best PJ (1989). Place cells and silent cells in the hippocampus of freely-behaving rats. J. Neurosci..

[21] Le Roux N (2013). Input-specific learning rules at excitatory synapses onto hippocampal parvalbumin-expressing interneurons. J. Physiol..

[22] Matta JA (2013). Developmental origin dictates interneuron AMPA and NMDA receptor subunit composition and plasticity. Nat. Neurosci..

[23] Ma J, Leung LS (2000). Relation between hippocampal gamma waves and behavioral disturbances induced by phencyclidine and methamphetamine. Behav. Brain Res..

[24] Korotkova T (2010). NMDA receptor ablation on parvalbumin-positive interneurons impairs hippocampal synchrony, spatial representations, and working memory. Neuron.

[25] Riebe I (2016). Tonically active NMDA receptors--a signalling mechanism critical for interneuronal excitability in the CA1 stratum radiatum. Eur. J. Neurosci..

[26] Grunze HC (1996). NMDA-dependent modulation of CA1 local circuit inhibition. J. Neurosci..

[27] von Engelhardt J (2015). GluN2D-containing NMDA receptors-mediate synaptic currents in hippocampal interneurons and pyramidal cells in juvenile mice. Front Cell Neurosci..

[28] Kotermanski SE, Johnson JW (2009). Mg2+ imparts NMDA receptor subtype selectivity to the Alzheimer's drug memantine. J. Neurosci..

[29] Hagino Y (2010). Essential role of NMDA receptor channel epsilon4 subunit (GluN2D) in the effects of phencyclidine, but not methamphetamine. PLoS One.

[30] Yamamoto H (2013). Involvement of the N-methyl-D-aspartate receptor GluN2D subunit in phencyclidine-induced motor impairment, gene expression, and increased Fos immunoreactivity. Mol Brain.

[31] Yamamoto T (2016). Role of the NMDA receptor GluN2D subunit in the expression of ketamine-induced behavioral sensitization and region-specific activation of neuronal nitric oxide synthase. Neurosci. Lett..

[32] Sapkota K (2016). GluN2D N-methyl-D-aspartate receptor subunit contribution to the stimulation of brain activity and gamma oscillations by ketamine: implications for schizophrenia. J. Pharmacol. Exp. Ther..

[33] Jentsch JD (1997). Enduring cognitive deficits and cortical dopamine dysfunction in monkeys after long-term administration of phencyclidine. Science.

[34] Slifstein M (2015). Deficits in prefrontal cortical and extrastriatal dopamine release in schizophrenia: a positron emission tomographic functional magnetic resonance imaging study. JAMA Psychiatry.

[35] Jentsch JD, Roth RH (1999). The neuropsychopharmacology of phencyclidine: from NMDA receptor hypofunction to the dopamine hypothesis of schizophrenia. Europsychopharmacology.

[36] Bubenikova-Valesova V (2008). Models of schizophrenia in humans and animals based on inhibition of NMDA receptors. Neurosci. Biobehav. Rev..

[37] Janhunen SK (2015). The subchronic phencyclidine rat model: relevance for the assessment of novel therapeutics for cognitive impairment associated with schizophrenia. Psychopharmacology (Berl).

[38] Dalmau J (2011). Clinical experience and laboratory investigations in patients with anti-NMDAR encephalitis. Lancet Neurol..

[39] Titulaer MJ (2013). Treatment and prognostic factors for long-term outcome in patients with anti-NMDA receptor encephalitis: an observational cohort study. Lancet Neurol..

[40] Dalmau J (2008). Anti-NMDA-receptor encephalitis: case series and analysis of the effects of antibodies. Lancet Neurol..

[41] Moscato EH (2014). Acute echanisms underlying antibody effects in anti-N-methyl-D-aspartate receptor encephalitis. Ann. Neurol..

[42] Nyiri G (2003). Large variability in synaptic N-methyl-D-aspartate receptor density on interneurons and a comparison with pyramidal-cell spines in the rat hippocampus. Neuroscience..

[43] Masdeu JC, Dalmau J, Berman KF (2016). NMDA receptor internalization by autoantibodies: a reversible mechanism underlying psychosis?. Trends Neurosci..

[44] Gresa-Arribas N (2014). Antibody titres at diagnosis and during follow-up of anti-NMDA receptor encephalitis: a retrospective study. Lancet Neurol..

[45] Weinberger DR (1996). On the plausibility of "the neurodevelopmental hypothesis" of schizophrenia. Neuropsychopharmacology..

[46] Deutsch SI, Mastropaolo J, Rosse RB (1998). Neurodevelopmental consequences of early exposure to phencyclidine and related drugs. Clin. Neuropharmacol..

[47] Brown AS, Derkits EJ (2010). Prenatal infection and schizophrenia: a review of epidemiologic and translational studies. Am. J. Psychiatry.

[48] Paule MG (2011). Ketamine anesthesia during the first week of life can cause long-lasting cognitive deficits in rhesus monkeys. Neurotoxicol. Teratol..

[49] du Bois TM, Huang XF (2007). Early brain development disruption from NMDA receptor hypofunction: relevance to schizophrenia. Brain Res. Rev..

[50] Lim AL, Taylor DA, Malone DT (2012). Consequences of early life MK-801 administration: long-term behavioural effects and relevance to schizophrenia research. Behav. Brain Res..

[51] Rujescu D (2006). A pharmacological model for psychosis based on N-methyl-D-aspartate receptor hypofunction: molecular, cellular, functional and behavioral abnormalities. Biol. Psychiatry.

[52] Abekawa T (2007). Prenatal exposure to an NMDA receptor antagonist, MK-801 reduces density of parvalbumin-immunoreactive GABAergic neurons in the medial prefrontal cortex and enhances phencyclidine-induced hyperlocomotion but not behavioral sensitization to methamphetamine in postpubertal rats. Psychopharmacology (Berl).

[53] Behrens MM (2007). Ketamine-induced loss of phenotype of fast-spiking interneurons is mediated by NADPH-oxidase. Science.

[54] Wang CZ (2008). Postnatal phencyclidine administration selectively reduces adult cortical parvalbumin-containing interneurons. Neuropsychopharmacology.

[55] Coleman LG (2009). Deficits in adult prefrontal cortex neurons and behavior following early post-natal NMDA antagonist treatment. Pharmacol. Biochem. Behav..

[56] Kjaerby C (2014). Impaired GABAergic inhibition in the prefrontal cortex of early postnatal phencyclidine (PCP)-treated rats. Cereb. Cortex.

[57] Jeevakumar V (2015). Ketamine administration during the second postnatal week induces enduring schizophrenia-like behavioral symptoms and reduces parvalbumin expression in the medial prefrontal cortex of adult mice. Behav. Brain. Res..

[58] Jeevakumar V, Kroener S (2016). Ketamine administration during the second postnatal week alters synaptic properties of fast-spiking interneurons in the medial prefrontal cortex of adult mice. Cereb. Cortex.

[59] Contestabile A (2000). Roles of NMDA receptor activity and nitric oxide production in brain development. Brain Res. Brain Res. Rev..

[60] Komuro H, Rakic P (1993). Modulation of neuronal migration by NMDA receptors. Sci..

[61] Desfeux A (2010). Dual effect of glutamate on GABAergic interneuron survival during cerebral cortex development in mice neonates. Cereb. Cortex.

[62] Awobuluyi M, Lipton SA, Sucher NJ (2003). Translationally distinct populations of NMDA receptor subunit NR1 mRNA in the developing rat brain. J. Neurochem..

[63] Hestrin S (1992). Developmental regulation of NMDA receptor-mediated synaptic currents at a central synapse. Nature.

[64] Wang HX, Gao WJ (2009). Cell type-specific development of NMDA receptors in the interneurons of rat prefrontal cortex. Neuropsychopharmacology.

[65] Rotaru DC (2011). Glutamate receptor subtypes mediating synaptic activation of prefrontal cortex neurons: relevance for schizophrenia. J. Neurosci..

[66] Belforte JE (2010). Postnatal NMDA receptor ablation in corticolimbic interneurons confers schizophrenia-like phenotypes. Nat. Neurosci..

[67] Povysheva NV, Johnson JW (2012). Tonic NMDA receptor-mediated current in prefrontal cortical pyramidal cells and fast-spiking interneurons. J. Neurophysiol..

[68] Doischer D (2008). Postnatal differentiation of basket cells from slow to fast signaling devices. J. Neurosci..

[69] Goldberg EM (2011). Rapid developmental maturation of neocortical FS cell intrinsic excitability. Cereb. Cortex.

[70] Jones KS, Corbin JG, Huntsman MM (2014). Neonatal NMDA receptor blockade disrupts spike timing and glutamatergic synapses in fast spiking interneurons in a NMDA receptor hypofunction model of schizophrenia. PLoS. One.

[71] Owczarek S (2011). Phencyclidine treatment increases NR2A and NR2B N-methyl-D-aspartate receptor subunit expression in rats. Neuroreport.

[72] Forrest D (1994). Targeted disruption of NMDA receptor 1 gene abolishes NMDA response and results in neonatal death. Neuron.

[73] Li Y (1994). Whisker-related neuronal patterns fail to develop in the trigeminal brainstem nuclei of NMDAR1 knockout mice. Cell.

[74] Elberger AJ, Deng J (2003). Corpus callosum and visual cortex of mice with deletion of the NMDA-NR1 receptor: I. Accelerated development of callosal projection neurons. Brain. Res. Dev. Brain Res..

[75] Woodruff PW, McManus IC, David AS (1995). Meta-analysis of corpus callosum size in schizophrenia. J. Neurol. Neurosurg. Psychiatry.

[76] Arnone D (2008). Meta-analysis of magnetic resonance imaging studies of the corpus callosum in schizophrenia. Schizophr. Res..

[77] Mohn AR (1999). Mice with reduced NMDA receptor expression display behaviors related to schizophrenia. Cell.

[78] Gainetdinov RR, Mohn AR, Caron MG (2001). Genetic animal models: focus on schizophrenia. Trends. Neurosci..

[79] Moy SS (2012). Preweaning sensorimotor deficits and adolescent hypersociability in Grin1 knockdown mice. Dev. Neurosci..

[80] Barkus C (2012). GluN1 hypomorph mice exhibit wide-ranging behavioral alterations. Genes Brain Behav..

[81] Bodarky CL (2009). Novel environment and GABA agonists alter event-related potentials in N-methyl-D-aspartate NR1 hypomorphic and wild-type mice. J. Pharmacol. Exp. Ther..

[82] Gandal MJ (2012). Mice with reduced NMDA receptor expression: more consistent with utism than schizophrenia?. Genes Brain Behav..

[83] Humphries C (1996). NMDA receptor mRNA correlation with antemortem cognitive impairment in schizophrenia. Neuroreport.

[84] Sokolov BP (1998). Expression of NMDAR1, GluR1, GluR7, and KA1 glutamate receptor mRNAs is decreased in frontal cortex of "neuroleptic-free" schizophrenics: evidence on reversible up-regulation by typical neuroleptics. J. Neurochem..

[85] Gao XM (2000). Ionotropic glutamate receptors and expression of N-methyl-D-aspartate receptor subunits in subregions of human hippocampus: effects of schizophrenia. Am. J. Psychiatry.

[86] Ibrahim HM (2000). Ionotropic glutamate receptor binding and subunit mRNA expression in thalamic nuclei in schizophrenia. Am. J. Psychiatry.

[87] Law AJ, Deakin JF (2001). Asymmetrical reductions of hippocampal NMDAR1 glutamate receptor mRNA in the psychoses. Neuroreport.

[88] Beneyto M, Meador-Woodruff JH (2008). Lamina-specific abnormalities of NMDA receptor-associated postsynaptic protein transcripts in the prefrontal cortex in schizophrenia and bipolar disorder. Neuropsychopharmacology.

[89] Weickert CS (2013). Molecular evidence of N-methyl-D-aspartate receptor hypofunction in schizophrenia. Mol. Psychiatry.

[90] Akbarian S (1996). Selective alterations in gene expression for NMDA receptor subunits in prefrontal cortex of schizophrenics. J. Neurosci..

[91] Dracheva S (2001). N-methyl-D-aspartic acid receptor expression in the dorsolateral prefrontal cortex of elderly patients with schizophrenia. Am. J. Psychiatry.

[92] Kristiansen LV (2007). NMDA receptors and schizophrenia. Curr. Opin. Pharmacol..

[93] Catts VS (2016). A quantitative review of the postmortem evidence for decreased cortical N-methyl-d-aspartate receptor expression levels in schizophrenia: How can we link molecular abnormalities to mismatch negativity deficits?. Biol. Psychol..

[94] Banerjee A (2015). Src kinase as a mediator of convergent molecular abnormalities leading to NMDAR hypoactivity in schizophrenia. Mol. Psychiatry..

[95] Iwasato T (2000). Cortex-restricted disruption of NMDAR1 impairs neuronal patterns in the barrel cortex. Nature..

[96] Ultanir SK (2007). Regulation of spine morphology and spine density by NMDA receptor signaling in vivo. Proc. Natl. Acad. Sci. USA.

[97] Quintero GC, Erzurumlu RS, Vaccarino AL (2008). Evaluation of morphine analgesia and motor coordination in mice following cortex-specific knockout of the N-methyl-D-aspartate NR1-subunit. Neurosci. Lett..

[98] Sawtell NB (2003). NMDA receptor-dependent ocular dominance plasticity in adult visual cortex. Neuron.

[99] Rompala GR (2013). Contribution of NMDA receptor hypofunction in prefrontal and cortical excitatory neurons to schizophrenia-like phenotypes. PLoS. One.

[100] Tatard-Leitman VM (2015). Pyramidal cell selective ablation of N-methyl-D-aspartate receptor 1 causes increase in cellular and network excitability. Biol. Psychiatry.

[101] Tsien JZ, Huerta PT, Tonegawa S (1996). The essential role of hippocampal CA1 NMDA receptor-dependent synaptic plasticity in spatial memory. Cell.

[102] Fukaya M (2003). Retention of NMDA receptor NR2 subunits in the lumen of endoplasmic reticulum in targeted NR1 knockout mice. Proc. Natl. Acad. Sci. USA.

[103] Jinde S (2009). Lack of kainic acid-induced gamma oscillations predicts subsequent CA1 excitotoxic cell death. Eur. J. Neurosci..

[104] Olney JW, Newcomer JW, Farber NB (1999). NMDA receptor hypofunction model of schizophrenia. J. Psychiatr. Res..

[105] Lisman JE (2008). Circuit-based framework for understanding neurotransmitter and risk gene interactions in schizophrenia. Trends. Neurosci..

[106] Gonzalez-Burgos G, Lewis DA (2012). NMDA receptor hypofunction, parvalbumin-positive neurons, and cortical gamma oscillations in schizophrenia. Schizophr. Bull..

[107] Nakazawa K (2012). GABAergic interneuron origin of schizophrenia pathophysiology. Neuropharmacology..

[108] Cohen SM (2015). The impact of NMDA receptor hypofunction on GABAergic neurons in the pathophysiology of schizophrenia. Schizophr. Res..

[109] Neske GT, Patrick SL, Connors BW (2015). Contributions of diverse excitatory and inhibitory neurons to recurrent network activity in cerebral cortex. J. Neurosci..

[110] Standaert DG (1996). Expression of NMDAR2D glutamate receptor subunit mRNA in neurochemically identified interneurons in the rat neostriatum, neocortex and hippocampus. Brain. Res. Mol. Brain. Res..

[111] Yamasaki M (2014). Opposing role of NMDA receptor GluN2B and GluN2D in somatosensory development and maturation. J. Neurosci..

[112] Carlen, M., et al. A critical role for NMDA receptors in parvalbumin interneurons for gamma rhythm induction and behavior. *Mol. Psychiatry* **17**, 537–548 (2012). 10.1038/mp.2011.31 PMC3335079 21468034

[113] Cardin JA (2009). Driving fast-spiking cells induces gamma rhythm and controls sensory responses. Nature.

[114] Sohal VS (2009). Parvalbumin neurons and gamma rhythms enhance cortical circuit performance. Nature.

[115] Owens EM (2016). Electrophysiological Endophenotypes for Schizophrenia. Harv. Rev. Psychiatry.

[116] Saunders JA (2013). Knockout of NMDA receptors in parvalbumin interneurons recreates autism-like phenotypes. Autism Res..

[117] Monory K (2006). The Endocannabinoid System Controls Key Epileptogenic Circuits in the Hippocampus. Neuron.

[118] Jiang Z (2013). Social isolation exacerbates schizophrenia-like phenotypes via oxidative stress in cortical interneurons. Biol. Psychiatry.

[119] Nakao K, Nakazawa K (2014). Brain state-dependent abnormal LFP activity in the auditory cortex of a schizophrenia mouse model. Front. Neurosci..

[120] Chung, D.W., K.N. Fish & D.A. Lewis, Pathological basis for deficient excitatory drive to cortical parvalbumin interneurons in schizophrenia. *Am. J. Psychiatry* (2016).**173**, 1131–1139 (2016) 10.1176/appi.ajp.2016.16010025 PMC5089927 27444795

[121] Erhardt, S. *et al*. The kynurenine pathway in schizophrenia and bipolar disorder. *Neuropharmacology* **112**, 297–306 (2017). 10.1016/j.neuropharm.2016.05.020 27245499

[122] Herédi, J. et al. Astrocytic and neuronal localization of kynurenine aminotransferase-2 in the adult mouse brain. *Brain Struct. Funct.* (2016). doi:10.1007/s00429-016-1299-5. 10.1007/s00429-016-1299-5 27568378

[123] Campbell BM (2014). Kynurenines in CNS disease: regulation by inflammatory cytokines. Front. Neurosci..

[124] Bagasrawala I, Zecevic N, Radonjic N (2016). N-methyl D-aspartate receptor antagonist kynurenic acid affects human cortical development. Frontiers in Neuroscience.

[125] Korinek M (2011). Neurosteroid modulation of N-methyl-d-aspartate receptors: Molecular mechanism and behavioral effects. Steroids..

[126] Parizek A (2005). Neuroactive pregnanolone isomers during pregnancy. J. Clin. Endocrinol. Metab..

[127] Malayev A, Gibbs TT, Farb DH (2002). Inhibition of the NMDA response by pregnenolone sulphate reveals subtype selective modulation of NMDA receptors by sulphated steroids. Br. J. Pharmacol..

[128] Vales K (2012). 3alpha5beta-Pregnanolone glutamate, a use-dependent NMDA antagonist, reversed spatial learning deficit in an animal model of schizophrenia. Behav. Brain. Res..

[129] Vullhorst D (2015). A negative feedback loop controls NMDA receptor function in cortical interneurons via neuregulin 2/ErbB4 signalling. Nat Commun.

[130] Chung DW (2016). Dysregulated ErbB4 Splicing in Schizophrenia: Selective Effects on Parvalbumin Expression. Am. J. Psychiatry..

[131] Hahn CG (2006). Altered neuregulin 1-erbB4 signaling contributes to NMDA receptor hypofunction in schizophrenia. Nat. Med..

[132] Murray JD (2014). Linking microcircuit dysfunction to cognitive impairment: effects of disinhibition associated with schizophrenia in a cortical working memory model. Cereb. Cortex..

[133] Bygrave AM (2016). Knockout of NMDA-receptors from parvalbumin interneurons sensitizes to schizophrenia-related deficits induced by MK-801. Transl Psychiatry.

[134] Zweifel LS (2009). Disruption of NMDAR-dependent burst firing by dopamine neurons provides selective assessment of phasic dopamine-dependent behavior. Proc. Natl. Acad. Sci. U. S. A..

[135] Lisman J (2012). Excitation, inhibition, local oscillations, or large-scale loops: what causes the symptoms of schizophrenia?. Curr. Opin. Neurobiol..

[136] Pratt JA, Morris BJ (2015). The thalamic reticular nucleus: a functional hub for thalamocortical network dysfunction in schizophrenia and a target for drug discovery. J. Psychopharmacol..

[137] Vukadinovic Z (2014). NMDA receptor hypofunction and the thalamus in schizophrenia. Physiol. Behav..

[138] Nong Y, Huang Y-Q, Salter MW (2004). NMDA receptors are movin’ in. Curr. Opin. Neurobiol..

[139] Pérez-Otaño I, Ehlers MD (2005). Homeostatic plasticity and NMDA receptor trafficking. Trends. Neurosci..

[140] Alvarez VA, Ridenour DA, Sabatini BL (2007). Distinct structural and ionotropic roles of NMDA receptors in controlling spine and synapse stability. J. Neurosci..

[141] McKinney RA (2010). Excitatory amino acid involvement in dendritic spine formation, maintenance and remodelling. J. Physiol..

[142] Schizophrenia Working Group of the Psychiatric Genomics, C. (2014). Biological insights from 108 schizophrenia-associated genetic loci. Nature.

[143] Watkins CC, Andrews SR (2016). Clinical studies of neuroinflammatory mechanisms in schizophrenia. Schizophr. Res..

[144] Hardingham GE, Do KQ (2016). Linking early-life NMDAR hypofunction and oxidative stress in schizophrenia pathogenesis. Nat. Rev. Neurosci..

[145] Steullet P (2006). Synaptic plasticity impairment and hypofunction of NMDA receptors induced by glutathione deficit: relevance to schizophrenia. Neuroscience.

[146] Baxter PS (2015). Synaptic NMDA receptor activity is coupled to the transcriptional control of the glutathione system. Nat Commun..

[147] Cabungcal JH (2013). Early-life insults impair parvalbumin interneurons via oxidative stress: reversal by N-acetylcysteine. Biol. Psychiatry.

[148] Tamminga CA, Zukin RS (2015). Schizophrenia: evidence implicating hippocampal GluN2B protein and REST epigenetics in psychosis pathophysiology. Neuroscience..

